# Importance of scattered white spots detected in the duodenum and their relationship with zonulin

**DOI:** 10.12669/pjms.40.11.9800

**Published:** 2024-12

**Authors:** Semih Sezer, Selim Demirci

**Affiliations:** 1 Semih Sezer, Department of Gastroenterology, Dr. Abdurrahman Yurtaslan Oncology Training and Research Hospital, Ankara, Turkey; 2 Selim Demirci, Department of Gastroenterology, Dr. Abdurrahman Yurtaslan Oncology Training and Research Hospital, Ankara, Turkey

**Keywords:** Leaky gut, Tigh junction, Scattered white spots, Duodenum, Intestinal lymphangiectasia

## Abstract

**Objective:**

Scattered white spots (SWS) seen in the duodenum during esophagogastroduodenoscopy are rare lesions. The histopathologic examination of SWS lesions reveals normal duodenal mucosa (ND), chronic nonspecific duodenitis, and intestinal lymphangiectasia (IL). The intestinal epithelium, through its barrier function, is responsible for tightly controlling antigen traffic from the intestinal lumen to the submucosa. Zonulin plays a crucial role in intestinal barrier function. In this study, we aimed to determine whether there is a relationship between zonulin, the most important marker of leaky gut syndrome, and SWS lesions associated with lymphatic stasis and inflammation in the duodenum.

**Methods:**

This cross-sectional study at Dr. Abdurrahman Yurtaslan Oncology Hospital in Turkey, conducted from September to December 2023, included 63 patients with SWS and 30 control patients with ND. SWS lesions were categorized into two groups, Grade-1 and Grade-2, based on the degree of density and endoscopic appearance. Biopsies were taken from the duodenum, antrum, and corpus. Blood samples were taken for serum zonulin levels.

**Results:**

The difference between zonulin values was not significant in the control and patient groups (p>0.05). Metaplasia in the antrum was significantly higher in the SWS group than in the control group (p<0.05). There was no relationship between duodenal biopsy results and zonulin values (p>0.05).

**Conclusions:**

Patients with identified SWS more frequently exhibit IL and gastric metaplasia. There is no relationship between SWS and serum zonulin levels.

## INTRODUCTION

The duodenum is a very important part of the small intestine. It is susceptible to various diseases caused by systemic and local infectious agents, the use of nonsteroidal anti-inflammatory drugs, Crohn’s disease, celiac disease, Whipple’s disease, eosinophilic gastroenteritis, lymphangiectasia, and others.^1^ The duodenum plays a critical role in preventing the intracellular entry of foreign antigens or toxins.^2^,^3^ Tight junctions (TJs) in the intestinal epitelium tightly regulate paracellular antigen transport. In this fine-tuned system, the human protein zonulin plays a crucial role in controlling TJ and altering intestinal permeability. The system can be influenced by genetic or environmental factors (celiac disease and various pathogenic bacteria), allowing paracellular passage of intestinal pathogens and allergens.^4^-^8^ An increase in serum zonulin levels is recognized as a biological indicator of disrupted intestinal permeability. Measuring zonulin levels plays a crucial role in evaluating gut barrier function and understanding the pathophysiology of gut-related diseases.^9^

**Fig.1:**
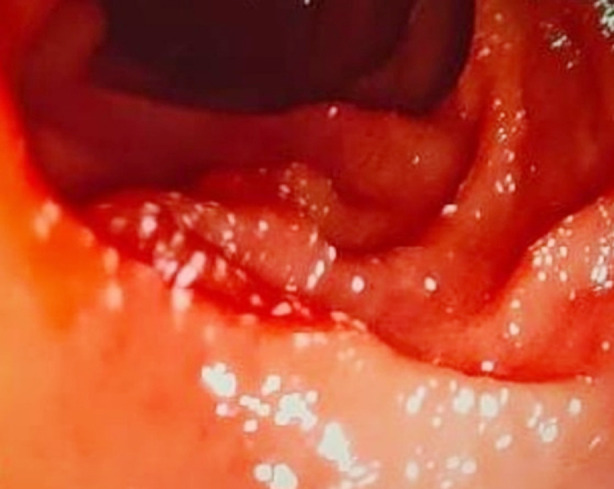
Scattered White Spots Grade-1.

**Fig.2:**
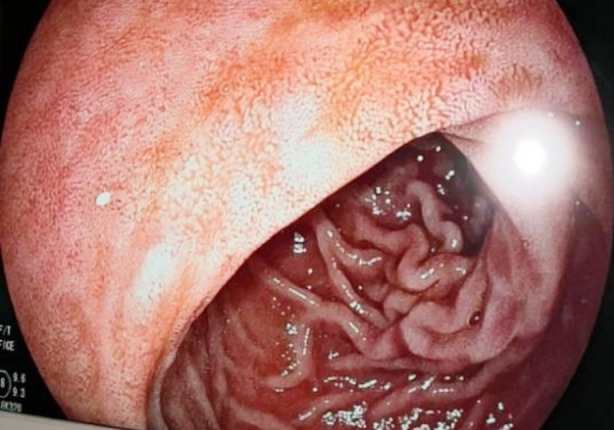
Scattered White Spots Grade-2.

During esophagogastroduodenoscopy (EGD), we occasionally encounter lesions in the duodenum that appear as diffuse scattered white spots (SWS) on the normal mucosa (Fig. 1 and 2). In previous studies, the prevalence of SWS was found as 1% and 3.2%. Although it is not fully understood in terms of its specific relationship and clinical significance with any specific disease, chronic non-specific duodenitis (CND) and intestinal lymphangiectasia (IL) have often been observed in lesions with SWS in some studies.^10^,^11^

In our study, we aimed to investigate the relationship between lesions and SWS appearance in the duodenum associated with lymphatic stasis and inflammation and zonulin, which plays an important role in intestinal barrier function.

## METHODS

Between September 2023 and December 2023, a total of 1024 patients who underwent EGD at the Dr. Abdurrahman Yurtaslan Oncology Training and Research in Turkey were included in this cross-sectional study. ClinicalTrials registration number: NCT06200129. The research was conducted in accordance with the principles of the Helsinki Declaration. Written and verbal informed consent was obtained from all patients.

### Ethical Approval:

Ethics committee approval was received for the research (Date: 06.09.2023, Decision No: 2023-08/333).

The sample size was determined according to Hertzog’s power analysis suggestion.^12^ Patients over 18 years of age were included in the study. Control group was formed from patients with normal duodenal mucosa on EGD, and patient group was formed from patients with SWS lesions. Patients with SWS were classified as Grade-1 and Grade-2 according to SWS density. Grade-1 (Fig.1) was defined as lesions that appeared as white spots on normal duodenal mucosa and could not be erased by washing, and Grade-2 (Fig.2) was defined as a diffuse or patchy white appearance on villi that could not be erased by washing. Of the 63 patients with SWS, 32 had a Grade-1 appearance and 31 had Grade-2 appearance. For the control group, 30 patients were included. Individuals with celiac disease, acute-chronic renal failure, duodenal ulcer, duodenal tumor were excluded.

### Endoscopic procedure:

An endoscopic examination was performed after at least 12 hours of fasting. Written consent was obtained from the patients before endoscopy. EGD was performed using a forward-thinking video gastroscope (Fujinon EG-590 WR, FUJINON Corporation, Saitama, Japan) by two experienced endoscopists. The control group patients were selected from among the patients who requested endoscopy for similar reasons as the patient group, had similar exclusion criteria and did not show SWS appearance in the second part of the duodenum. In both the patient and control groups, at least three biopsies were taken from the antrum, three from the corpus, and three from the second part of the duodenum. Biopsy samples were placed in 10% formalin and then embedded in paraffin. After staining with hematoxylin and eosin (H&E), the sections were examined by two expert pathologists for pathogens, intraepithelial lymphocytes, inflammation of the lamina propria, and villus structure. CND was defined as inflammation and leukocyte infiltration accompanied by edema. Intraepithelial lymphocytosis (IEL) was defined as the presence of more than 40 IELs per 100 epithelial cells in the small intestine, and IL was defined as the enlargement of the lymphatic ducts. Helicobacter pylori (Hp) was detected in all patients participating in the study by examining the biopsies taken from the antrum and corpus using both H&E staining and Giemsa staining.^13^-^16^

### Laboratory measurements and the zonulin test procedure:

The diagnosis of leaky gut syndrome was defined as serum zonulin levels above the normal value. Approximately 5 cc of blood was drawn from the antecubital veins of patients to measure serum zonulin levels. After clotting at room temperature for one hour and 30 minutes, the blood was centrifuged at 2000 revolutions per minute for 15 minutes using an Yuda 800D brand centrifuge. The obtained serum samples were stored at -80 degrees Celsius in a Thermo brand freezer for a period of three months. Zonulin levels in the serum samples were measured using commercial BT-LAB Human Zonulin ELISA kits (Cat.No: E1117Hu) (Shanghai Crystal Day Biotech Co., Ltd, Shanghai, China). The enzymatic reactions were measured photometrically. Zonulin values were determined by comparing the optical density of serum samples with the standard curve. The assay range of the kit was 2-600ng/mL. The sensitivity of test was 1,09ng/mL. The most appropriate curve was drawn through the points on the graph. Consequently, serum zonulin levels above 12ng/ml were considered positive. Albumin (g/dL) was measured in serum using a colorimetric assay with bromocresol green (AU5800- autoanalyzer, Beckman Gulter). In our study, low serum albumin level was defined as ≤4.0 g/dL.^17^

### Statistical analysis:

Descriptive statistics for continuous variables included mean, standard deviation, median, minimum, maximum, and interquartile range (IQR), while discrete variables were presented as counts and percentages. The normal distribution of the data was assessed using the Shapiro-Wilk test. One-way analysis of variance (ANOVA) was employed for comparisons between white spot groups in continuous variables that showed a normal distribution, and post-hoc analysis was conducted using the Tukey test to identify specific group differences. For continuous variables that did not follow a normal distribution, the Kruskal-Wallis variance analysis was applied, and the source of differences between groups was further examined using the Kruskal-Wallis test.

An independent sample t-test or Mann-Whitney U test was used for comparisons between two groups for continuous variables, depending on the normality of the data. The Chi-square/Fisher’s exact test was utilized for group comparisons of nominal variables (in cross-tabulations). IBM SPSS for Windows 20.0 (SPSS Inc., Chicago, IL) was used for statistical analysis, and a significance level of p<0.05 was considered.

## RESULTS

**Fig.3:**
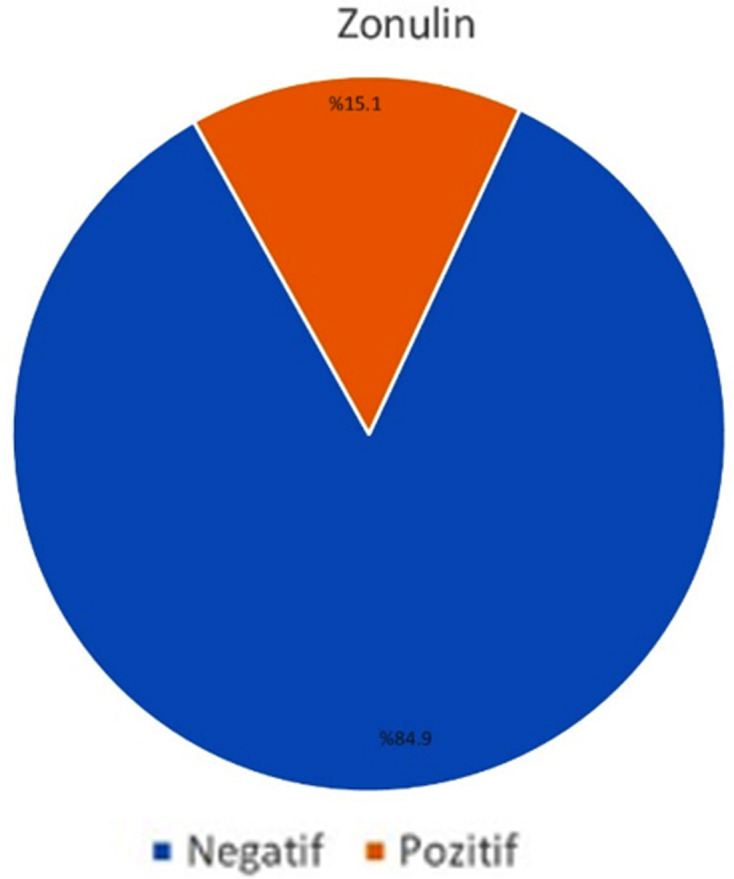
Zonulin value.

Demographic data for all patients. Table-I. Of the patients, 51.6% were female, 48.4% were male, with a mean age of 51.75±15.23 years, and the minimum and maximum ages were 18 and 80 years, respectively. The average body mass index (BMI) of the groups was similar (p>0.05). In 15.1% of the patients, zonulin levels were found to be positive (Fig.3). There was no significant difference in the positivity of serum zonulin values between the SWS groups and the control group (p>0.05). Albumin levels were found to be lower in SWS Grade 1 and 2 patients compared to the control group (p=0.007).

**Table-I:** Demographic characteristics and laboratory results of the patients.

	Control (n = 30)	SWS-Grade-1 (n = 32)	SWS-Grade-2 (n = 31)	p
Age (year):				
Mean (SD)	47.23 (16.32)	57.25 (13.52)	50.45 (14.51)	0.028 ^a^
BMI (kg/m^2^):				
Mean (SD)	26.97 (4.90)	27.12 (4.59)	27.10 (5.09)	0.991 ^a^
Sex: n (%)				
Female	14 (46.7)	18 (56.2)	16 (51.6)	0.272 ^c^
Male	16 (53.3)	14 (43.8)	15 (48.4)	
Laboratory test:				
Albumin (g/L)				
Mean (SD)	42.36 (2.17)	41.15 (1.95)	40.80 (2.07)	0.007^a^
Zonulin(ng/ml)				
Mean (SD) Median (IQR)	7.80 (13.89) 2.09 (1.64-3.60)	9.15 (15.26) 2.31 (1.82-4.91)	5.58 (9.11) 2.10 (1.83-3.34)	0.623^b^
Zonulin Values Positive (>12ng/ml) n (%)	5 (16.7)	7 (21.9)	5 (16.1)	0.809^c^

Values are presented as the mean ± SD, median (25 %-75 % interquartiles), and number (%). Statistically significant values are marked as bold. SWS: Scattered white spots; BMI: Body mass index; IQR: interquartile range; SD: standard deviation; n: number of patients a; One-way Analysis of Variance, b: Kruskal-Wallis Variance Analysis, c: Chi-Square test/Fisher’s Exact test.

The histopathologic examination results for the patient groups. Table-II. In the SWS group, the rate of duodenal lymphangiectasia was more frequently observed compared to the control group (25% and 22.6% versus 0%). In the control group, the rate of normal histopathologic examination of duodenal mucosa (ND) was more frequently observed compared to patients with SWS (63.3% versus 43.8% and 38.7%, respectively). The rate of intestinal metaplasia in the antrum was higher in SWS Grade-1 and SWS Grade-2 patients compared to the control group (43.8% and 22.6% versus 10%).

**Table-II:** Biopsy results of the antrum, corpus, and duodenum in patients with normal duodenum and SWS.

	Control	SWS-Grade-1	SWS-Grade-2	p

	n (%)	n (%)	n (%)	
Duodenal Biopsy:				
Non-specific duodenitis	11 (36.7)	10 (31.3)	12 (38.7)	0.048^c^
Duodenal lymphangiectasia	0 (0)	8 (25.0)	7 (22.6)	
Normal duodenal mucosa	19 (63.3)	14 (43.8)	12 (38.7)	
Gastric Biopsy:				
Antrum located Hp positive	13 (43.3)	14 (43.8)	11 (35.5)	0.541 ^c^
Corpus located Hp positive	13 (43.3)	16 (50.0)	10 (32.3)	0.355 ^c^
Antrum located intestinal metaplasia	3 (10.0)	14 (43.8)	7 (22.6)	0.009 ^c^
Corpus located intestinal metaplasia	2 (6.7)	7 (21.9)	7 (22.6)	0.177^c^

Values are presented as the number (%). Statistically significant values are marked as bold. n: number of patients; Hp: Helicobacter pylori; SWS: Scattered white spots, c: Chi -Square test/Fisher's Exact test.

The relationship between the pathology findings of biopsies taken from the stomach and duodenum and serum zonulin levels are shown in Table-III. There was no significant difference in the presence of Hp and intestinal metaplasia in the antrum and corpus regarding serum zonulin values (p>0.05 as well as between patients’ duodenal pathology results and serum zonulin levels (p>0.05).

**Table-III:** Comparison of gastric and duodenal biopsy results with serum zonulin levels.

	Zonulin Positive(>12 ng/ml)	p

	n (%)	
Gastric Biopsy		
Antrum Hp	10 (18.2)	0.977 ^c^
Antrum intestinal metaplasia	3 (12.5)	0.545 ^c^
Corpus Hp	8 (20.5)	0.636^c^
Corpus intestinal metaplasia	3 (18.8)	1.000^c^
Duodenal Biopsy		
Non-specific duodenitis	9 (27.3)	0.186^c^
Duodenal lymphangiectasia	1 (6.7)	
Normal duodenal mucosa	7 (15.6)	

Values are presented as the number (%). n: number of patients; Hp: Helicobacter pylori, c: Chi -Square test/Fisher’s Exact test.

## DISCUSSION

In our study, no significant difference in zonulin levels was found between the SWS and control groups. However, patients with SWS Grade-1 and Grade-2 lesions had a notably higher prevalence of duodenal lymphangiectasia compared to those without SWS (25% and 22.6% vs. 0%). Additionally, the rate of intestinal metaplasia in the antrum was higher in patients with SWS Grade-1 and Grade-2 compared to those without SWS (43.8% and 22.6% vs. 10%). The protein zonulin, an analogue of zonula occludens toxin, regulates intestinal TJ permeability through protein kinase C α-mediated actin polymerization.^18^ Increased intestinal permeability can allow toxins, microorganisms, and undigested food particles to enter the bloodstream, leading to inflammation and potentially triggering autoimmune diseases. Therefore, serum zonulin levels are considered an important biomarker for assessing intestinal barrier dysfunction.^19^

In recent years, studies on zonulin, which plays an important role in leaky gut syndrome, have been increasing. Previous studies have found an increased frequency of autoimmune disease and intra-abdominal malignancy in individuals with leaky gut syndrome and SWS lesions.^20^ The potential involvement of similar etiologies in both SWS and leaky gut syndrome prompted us to investigate whether there is a relationship between these two pathological conditions. Bıyıkoğlu *et al*.^10^ examined the biopsies of 107 patients with SWS in the duodenum and found IL in 36.4%, CND in 28.1%, giardiasis in 14%, and villous atrophy in 3.7%. In a study evaluating SWS lesions in the small intestine, Bellutti et al.^21^ found that these lesions were secondary to dilatation of the lymphatic vessels. IL is a rare condition characterized by the enlargement of intestinal lymphatics. This condition can lead to hypoproteinemia, edema, and hypogammaglobulinemia.^6^ In their study, Patel *et al*.^22^ showed that incidental lymphangiectasia can occur without malabsorption and did not observe any clinical symptoms in these patients.

In our study, in contrast to Bıyıkoğlu *et al.*, we did not identify any instances of giardiasis or celiac disease. Similarly, while clinical lymphangiectasia was not observed in our patients, the albumin level in the SWS group was found to be lower compared to the control group. This situation can be explained, as suggested by Patel *et al.*, by incidental lymphangiectasia in the SWS group. Taş et al.^11^ observed CND in 73.3% and IL in 44.3% of biopsy specimens obtained from SWS lesions. They found a significant reduction in SWS lesions in the duodenum with Hp eradication treatment. In our study, we found no correlation between Hp positivity and SWS lesions, whereas we found a correlation with intestinal metaplasia in the antrum. In our study, IL was not observed in the control group, while it was significantly higher in the SWS groups (23.8%). This supports the association of SWS lesions with IL. In contrast to Taş et al.^11^, we found a significant association between SWS lesions and intestinal metaplasia in the antrum. Our comparison between the group with SWS lesions and the control group with normal duodenal mucosa may have contributed to identifying this relationship. We did not find any previous studies in the literature demonstrating this association. This could be explained by the etiological factors causing SWS lesions also serving as predisposing factors for intestinal metaplasia.

### Strength of the study:

The strengths of our study include its distinction as the first investigation into the relationship between SWS and zonulin. In this context, it significantly contributes to the literature by providing deeper insights into the pathophysiological mechanisms of SWS. Additionally, we demonstrate a notably high prevalence of duodenal lymphangiectasia in patients with SWS Grade-1 and Grade-2 lesions, offering a new perspective on the clinical features of SWS. Furthermore, the increase in intestinal metaplasia rates in the antrum presents important implications for clinical practice regarding SWS.

### Limitations:

It includes the fact that the primary focus of the study was zonulin levels in SWS lesions, and diagnostic parameters for IL such as serum Ig levels, abdominal imaging, and alpha-1 antitrypsin levels in feces were not investigated. However, none of the patients had any indication for endoscopy related to hypoalbuminemia, edema, or protein-losing enteropathy. Another limitation is the lack of a classification system for SWS lesions with predetermined intensity and size. Also, we only measured zonulin levels in the serum. It would be useful to compare this with duodenal biopsy supernatant and fecal zonulin levels. Due to technical and financial limitations, these additional assessments have not been conducted.

## CONCLUSION

In our study, we did not find a correlation between SWS lesions and leaky gut syndrome. However, we observed an increased prevalence of intestinal lymphangiectasia and intestinal metaplasia in the antrum among patients with SWS lesions. To explore this topic more comprehensively, there is a need for larger, multicenter clinical studies with broader sample sizes.

## Data Availability:

All relevant data are within the paper and its supporting information files.

### Author Contributions:

**SS:** Study concept and design, conceptualization, methodology, statistical analysis, writing - original draft, performing endoscopic procedures, literature search.

**SD:** Performing endoscopic procedures, improve the language of the manuscript, and data collection.

All authors have read the final version and are responsible for the integrity of the study.
